# A scoping review of healthcare effectiveness data and information set (HEDIS) substance use disorder measures

**DOI:** 10.1080/07853890.2024.2447413

**Published:** 2025-01-02

**Authors:** Oliver Ethan Goal, Isca Amanda, Cameron Adams, Maria Adela Grando

**Affiliations:** a College of Health Solutions, Arizona State University, Phoenix, Arizona, USA; b Arizona Health Care Cost Containment System, Phoenix, Arizona, USA

**Keywords:** Scoping review, HEDIS metrics, addiction

## Abstract

**Introduction:**

There is a need to assess the delivery of interventions to improve substance use disorder (SUD) treatment, as measured by the Healthcare Effectiveness Data and Information Set (HEDIS®) metrics. The goal was to characterize published articles reporting HEDIS® SUD measures and recommend future work on applying and investigating SUD HEDIS® metrics and their effect on SUD treatments.

**Materials and Methods:**

The PRISMA-ScR scoping review protocol was used to find published work and investigate the most common reported baseline characteristics, HEDIS® metric outcomes, and knowledge gaps. Peer-reviewed papers available through PubMed, Academic Search Premier, Elsevier/ScienceDirect, and Medline were searched up to August 14, 2022.

**Results:**

Twenty-eight articles were included after removing 92 duplications. Twenty-five articles were retrospective cohort studies, two were RCTs, and there was a mixed-method study. SUD metrics were studied in diverse settings, including ED, primary care, mental health care, and SUD specialty care. Twenty-seven papers utilized the Initiation and Engagement of Substance Use Disorder Treatment (IET) measure, and 13 had similar data sources, study populations, and authors. Eight papers presented IET results by substance used, primarily alcohol, cannabis, and opioids.

**Conclusions:**

More research is needed on the HEDIS® SUD metrics and their usefulness in informing SUD prevention and treatment, policy, and public health outcomes.

## Introduction

Substance Use Disorder (SUD) is a group of conditions involving dependence and abuse of substances associated with significant morbidity, societal costs, and growing prevalence [1,2]. SUD includes a broad range of substances, including alcohol, tobacco, and illicit and prescription medications [3,4]. In 2019, an estimated 20.4 million or 7.4% of individuals 12 years of age and older had SUD in the past year [5]. Of these, an estimated 14.5 million had Alcohol Use Disorder (AUD), and 8.3 million had SUD with illicit substances. Because of the prevalence of opioid use disorders, the United States Department of Health and Human Services (HHS) declared a public health emergency in 2017 [6].

A division of HHS, the Centers for Medicare and Medicaid Services (CMS), measures processes and health outcomes through standardized metrics. A Core Quality Measures Collaborative, a diverse group of leaders in the healthcare industry, is convened by a CMS-contracted Consensus Based Entity to select these performance measures from an array of measure stewards [7]. This entity sometimes modifies the standard methodology to reflect better CMS enrollees (e.g. CMS modified the ‘Deprescribing of Benzodiazepines in Older Adults’ to ‘Concurrent Use of Opioids and Benzodiazepines’ to reduce the fatal overdoses associated with the combination of these drugs) [8].

The National Committee for Quality Assurance’s (NCQA) Healthcare Effectiveness Data and Information Set (HEDIS^®^) measures are some of the most widely reported quality measures in the United States [9]. HEDIS^®^ measures are updated, added, or removed regularly [10]. The HEDIS^®^ measures cover the topic of SUD among other disease states and are an important window into the state of SUD treatment [11]. HEDIS^®^ measures help to monitor progress on SUD prevention and treatment in a world where investigation into SUD is limited by patient privacy and stigma [12–14].

The 2022 HEDIS^®^ measures related to SUD include Diagnosed Substance Use Disorder (DSU), Follow-Up After High-Intensity Care for Substance Use Disorder (FUI), Follow-Up After Emergency Department Visit for Substance use (FUA), Initiation and Engagement of Substance Use Disorder Treatment (IET), Use of Opioids at High Dosage (HDO), Use of Opioids from Multiple Providers (UOP), Risk of Continued Opioid Use (COU), Pharmacotherapy for Opioid Use Disorder (POD), Unhealthy Alcohol Use Screening and Follow-Up (ASF-E), and Deprescribing of Benzodiazepines in Older Adults (DBO).

The IET measure was developed by the Washington Circle and implemented by the NCQA HEDIS^®^ [15,16]. The measure has changed over time, focusing on the specification rather than the conceptualization. Changes include adding the Healthcare Common Procedure Coding System codes and extending the treatment time frame for engagement from 30 to 34 days following initiation [17,18]. NCQA expects to remove alcohol as a qualifying substance in 2024 [19].

There is a need to assess the delivery of interventions to improve SUD treatment, as measured by HEDIS^®^ metrics, to guide SUD care, research, investment, policies, and regulations. Our main objective was to perform a scoping review of the literature to characterize published articles reporting HEDIS^®^ SUD measures. The review investigated the most common reported baseline characteristics, HEDIS^®^ metric outcomes (such as IET results) and identified knowledge gaps. The goal was to use the study findings to recommend future work on applying and investigating SUD HEDIS^®^ metrics and their effect on SUD treatments.

## Method

This study is a scoping review on HEDIS^®^ measures related explicitly to SUD following guidelines from the Preferred Reporting Items for Systematic Review and Meta-Analyses extension for Scoping Review (PRISMA-ScR) [20]. The most reported baseline characteristics and knowledge gaps in selected articles were also identified.

### Consent process and approving IRB/ethics Committee

This research did not involve human subjects; therefore, no approval from IRB/Ethics committees was required.

### Data availability

The authors confirm that the data supporting the findings of this study are available within the article and its supplementary materials.

### Selection of HEDIS^®^ measures

Ninety-six 2022 HEDIS^®^ measures were screened for relevance to SUD [21,48]. Ten HEDIS^®^ measures of interest were identified, including four general SUD measures, four opioid measures, one alcohol measure, and one benzodiazepine measure. Appendix A provides descriptions of each of the measures per the HEDIS^®^ 2022 measurement year, with details on NQF endorsement and CMS use [11]. Appendix B provides a list of all the abbreviations used in this paper.

### Search strategy

A systematic search was performed between July 17, 2022, and August 14, 2022. The scholarly databases PubMed, Academic Search Premier, Elsevier/ScienceDirect, and Medline were used. Main search terms included terminology for the HEDIS^®^ measure description, primary disorder (Substance-Related Disorders, Substance Use Disorder, SUD, or Drug Use or Drug Abuse), and drug/substance classes related to selected HEDIS^®^ measures (Substance, Alcohol, Opioid, or Benzodiazepine).

There were no limits on the date, language, or study design of the publications. The search was limited to peer-reviewed literature. See Appendix C for details on the search terms used in each database. Search results were compiled and tracked in an Excel file.

### Selection criteria

Duplicate articles were removed from the search results. Articles were then screened by title and abstract according to the selection criteria by OG and AG. Inclusion criteria included being available as a full-text English article, being published in a peer-reviewed journal, and reporting on one or more SUD-related HEDIS^®^ measures. Following the screening, full-text articles for the remaining results were obtained, read entirely, and confirmed for eligibility using the same inclusion criteria. Periodicals, conference abstracts, short communications, reviews of other articles, and literature reviews were excluded due to a lack of new findings and insufficient reporting.

### Data extraction and analysis

Three reviewers participated in this stage. In the first review, OG and AG independently reviewed the details regarding the title, authors, journal, year of publication, relevant HEDIS^®^ measures of interest, study design, related articles (if applicable), sample size, time or period of investigation, primary objective, and primary findings. Other pertinent details regarding setting, target population, and interventions were also extracted from each selected article. IA categorized the study design according to Grimes and Schulz’s algorithm for classifying types of clinical research [22]. Patterns and trends were noted during the first review and applied during the second review of all selected papers by OG and IA. AG resolved the disagreements from OG and IA’s reviews.

Twenty-seven out of 28 included articles applied the IET measure. Therefore, a more in-depth analysis was conducted of those 27 studies. These papers were reviewed a third time by OG and IA to compare the year of the IET specification applied, initiation and engagement rates, study populations, and data sources. Initiation results for the inpatient setting were nearly 100% or not reported and therefore excluded from our analysis.

Most common reported baseline characteristics appearing in 50% or more of the included articles were further analyzed by OG to help guide future research. A critical appraisal of the quality of included studies and potential publication bias was not performed in this study.

## Results

### Scoping review

**Figure 1.**
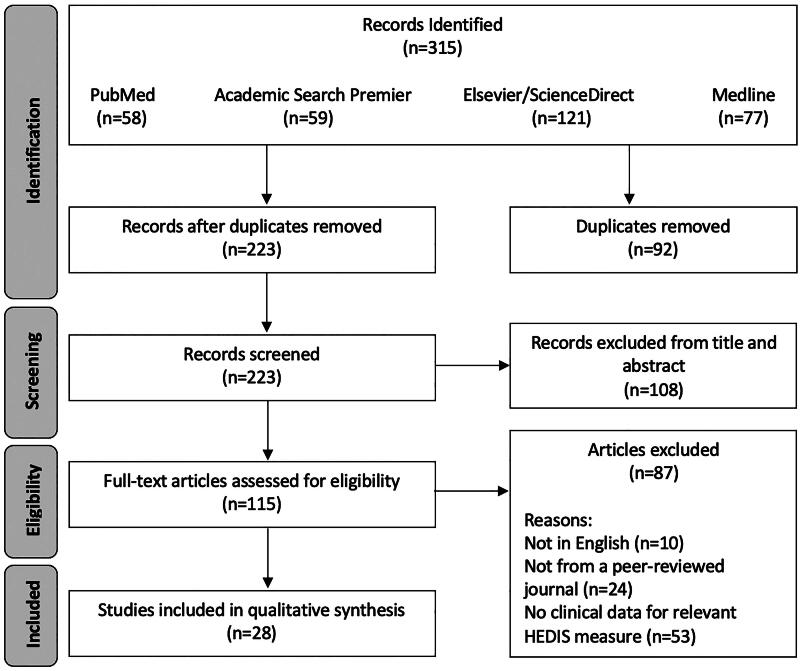
Flow Diagram of Scoping Review

A flow chart of the systematic literature search is depicted in Figure 1. We identified 315 records from the initial literature search, with 58 from PubMed, 59 from Academic Search Premier, 121 from Elsevier/ScienceDirect, and 77 from Medline (Appendix C).

We used the same keywords for PubMed and Medline searches. Fifty-three PubMed papers also appeared in the Medline search. Five PubMed papers did not appear in the Medline search. Fifty-three papers appeared in both the Medline and PubMed searches. Medline retrieved 24 papers that PubMed did not find.

After the removal of 92 duplicate records, 223 remained. Non-duplicated papers were screened by title and abstract, resulting in 115 records, with 108 excluded. Full-text articles for the 115 remaining records were assessed for eligibility, excluding 87 articles. Reasons for exclusion include not having a full-text English article (*n* = 10), not being from a peer-reviewed journal (*n* = 24), and not presenting clinical data on at least one HEDIS^®^ measure of interest (*n* = 53). This resulted in the 28 articles included in the review.

### Included publications

Details on included articles are displayed in chronological order in Table 1.

**Table 1.** Summary of papers on HEDIS SUD-related measures.

Author(s), Year	HEDIS Measure/s	Study Design	Sample Size	Male Gender	HEDIS SUD Finding(s)
Breton et al. [23]	IET	Retrospective cohort study	1,235	65%	13% of patients visiting the ED with a substance abuse diagnosis received follow-up substance abuse services within 14 days of the initial ED visit.
Harris et al. [24]	IET	Retrospective cohort study	320,238	Not Provided	32,728 patients were diagnosed in the outpatient setting with 7.3% engaging and 47,486 in the inpatient setting with 15.7% engaging.
Harris et al. [25]	IET	Retrospective cohort study	2,789	92.1%	The 1,463 out of 2,789 patients meeting the engagement measure improved significantly more in all domains compared to patients that did not engage.
Harris et al. [17]	IET	Retrospective cohort study	2,800	Not Provided	IET rates not reported.
MacLean et al. [26]	IET	Retrospective cohort study	5,198,592 (2,433,517 for HEDIS measures)	Not Provided	32.78% initiation rate and 11.42% engagement rate.
Bensley et al. [27]	IET	Retrospective cohort study	302,406	96%	17% initiation rate and 27% engagement rate.
Watkins et al. [28]	IET	Randomized Clinical Trial	377	79.6%	The proportion meeting HEDIS IET was higher with collaborative care vs usual care (Initiated: 31.6% vs 13.7%, *p* < 0.001; Engaged: 15.5% vs 4.2%, *p* < 0.001).
Urbanoski et al. [29]	IET	Retrospective cohort study	120,394	63.2%	About half of the clients that entered outpatient treatment met the criteria for initiation and 30% met engagement criteria.
Yarborough et al. [30]	IET	Mixed-Methods Study (Retrospective cohort study and Qualitative study/interview)	44,320	64.6% Commercial and 62.7% Medicare	Initiation rates of around 39.1% and 38.8 % and engagement rates of 13.6% and 3.1% for Commercial and Medicare plans.
Breslau et al. [31]	IET	Retrospective cohort study	378,289 (IET)	Not Provided	Initiation rates of 27.0%, 30.5%, 17.3%, and 15.6% as well as engagement rates of 2.4%, 2.4%, 1.7%, and 1.5% for Whites, Blacks, Hispanics, and Asians/Native Hawaiians/Other Pacific Islanders.
Setodji et al. [32]^a^	IET	Randomized Controlled Trial	258	76.0%	Initiation rate of 27.5% and engagement rate of 14.7%.
Binswanger et al. [33]	IET	Retrospective cohort study	86,565	60.4%	Initiation rate of 27.9% and engagement rate of 11.5%.
Campbell et al. [34]^b^	IET	Retrospective cohort study	11,490	46.9%	Initiation rate of 25.8% and 18.9%.
Garnick et al. [35]	IAD changed to DSU, IET	Retrospective cohort study	NSDUH: approximately 70,000 CMS MAX: Not specified	Not Reported	Eligible population prevalence rate was 10.0% with average identification rate at 2.9%. For the seven states investigated, the gap in prevalence and identification ranged from 5.1-11.0%, initiation rates ranged from 36.9%-57.1%, and engagement rates ranged from 11.8-31.1%. Initiation rate of 46.3% and engagement rate of 17.5%.
Hechter et al. [36]^b^	IET	Retrospective cohort study	86,565	60.4%	Initiation rate of 27.9% (11.4% excluding index episodes in the inpatient setting). AOD use disorder treatment initiation was similar in those with and without HIV diagnosis (10.3% vs 11.4%, *p* = 0.50). Of those that initiated, few engaged in both groups with and without HIV diagnosis (8.8% vs 11.5%, *p* = 0.31).
Kline-Simon et al. [37]^b^	IET	Retrospective cohort study	54,321	61.3%	Initiation rate of 9.3% and engagement rate of 17.1%.
Lapham et al. [38]	IET	Retrospective cohort study	15,202	63.1%	30.0% (95% CI: 29.2-30.7%) initiated, 6.9% (95% CI: 6.2-7.7%) engaged among the initiated, and 2.1% (95% CI: 1.9-2.3%) overall both initiated and engaged.
Lind et al. [39]	IET	Retrospective cohort study	2010: 198,505 2015: 689,565	55.6%	In the adolescent group, 48.5% initiation rate was reported in 2010 and 40.2% in 2015. In the adult group, 39.4% initiation was reported in 2010 and 34.6% in 2015. for the engagement rate, 36.1% was reported in 2010 and 26.0% in 2015 for the adolescent group. 24.0% engagement and 20.3% in 2015 was reported for adults.
Loree et al. [40]^b^	IET	Retrospective cohort study	86,565	60.4%	34.9% initiation rate and 10.3% engagement rate.
Weisner et al. [41]	IET	Retrospective cohort study	86,565	59.2%, 62.4%, 59.2%, 63.5%, 55.4%, 58.4%, 55.8% (by health system)	Overall initiation (27.9%) and engagement (11.5%) ha wide variation.
Englander et al. [42]	IET^d^	Retrospective cohort study	624	60.3%	Initiation rates were not reported. Only 17.2% of patients engaged in SUD treatment before hospitalization. IMPACT patients engaged in SUD treatment more often than controls following discharge (38.9% vs 23.3%, *p* < 0.01).
Adams et al. [43]	IET	Retrospective cohort study	338,708 Active duty and 178,801 National Guard/Reserve soldiers	93.7% (Active Duty), 90.7% (National Guard/ Reserve)	45.3% initiation rate and 25.0% engagement rate.
Gray et al. [44]	IET	Retrospective cohort study	4,726	94.8%	40% of soldiers initiated and 24% engaged.
Edmonds et al. [45]^c^	IET	Retrospective cohort study	299,455	95.9%	17.4% initiation rate and 27.1% engagement rate.
Regan et al. [46]	IET^d^	Retrospective cohort study	1,946	66%	25% of patients received referrals less often. 12% initiation rate and 8% engagement rate.
Martino et al. [47]	IET, HDO, UOP	Retrospective cohort study	198,464	Not Provided	HEDIS HDO results reported 95.0% for White and 99.2% for American Indian/Alaska Native beneficiaries. UOP for multiple prescribers reported 88.1% for White and 95.8% for American Indian/Alaska Native while UOP for multiple pharmacies reported 97.3% for White and 98.9% for American Indian/Alaska Native beneficiaries. 29.1% and 22.4% initiation rate were reported for Whites and American Indian/Alaska Natives. 3.3% and 2.0% engagement rates were reported for Whites and American Indian/Alaska Natives.
Chavez et al. [48]	IET	Retrospective cohort study	20,602	63.0%	49.5% of eligible adolescents initiated SUD treatment and 48.5% engaged.
Powell et al. [49]	FUA	Retrospective cohort study	69 (5 for HEDIS FUA)	31.9%	Follow-up care within 7 and 30 days of an ED visit for AOD was 20% and 60% for E-MHC patients, respectively.

AOD indicates Alcohol and Other Drug; aOR: adjusted Odds Ratio; AUD: Alcohol Use Disorder; CI: Confidence Interval; DSU: Diagnosed Substance Use Disorder; ED: Emergency Department; FUA: Follow-Up After Emergency Department Visit for Substance Use Disorder; HDO: Use of Opioids at High Dosage; HEDIS: Healthcare Effectiveness Data and Information Set; IAD: Identification of Alcohol and other Drug Services; IET: Initiation and Engagement of Substance Use Disorder Treatment; OR: Odds Ratio; RR: Relative Risk; SUD: Substance Use Disorder; SUPIC: Substance Use and Psychological Injury Combat; UOP: Use of Opioids from Multiple Providers; VA: Veterans Health Administration or Department of Veterans Affairs.

^a^ Uses results from Watkins et al. [28].

^b^ Uses results from Weisner et al. [41].

^c^ Uses results from Bensley et al. [27].

^d^Definition of Engagement is 34 days as opposed to 30 days.

Studies ranged in publication date from 2007 to 2022. Of the included articles, 27 reported on the Initiation and Engagement of Substance Use Disorder Treatment (IET) measure [23,27,31,33,34,42–45,48,25,17,24,26,28–30,32,36–41,46,47]. One article published in 2019 also reported on the Identification of Alcohol and other Drug Services (IAD) measure, which would later become the Diagnosed Substance Use Disorders (DSU) measure [35]. Another paper published in 2022 also reported on the Use of Opioids at High Dosage (HDO) and Use of Opioids from Multiple Providers (UOP) measures [47]. Finally, one article reported on the Follow-Up After Emergency Department Visit for Substance Use (FUA) measure [49]. No articles were found that reported on the Follow-Up After High-Intensity Care for Substance Use Disorder (FUI), Risk of Continued Opioid Use (COU), Pharmacotherapy for Opioid Use Disorder (POD), Unhealthy Alcohol Use Screening and Follow-Up (ASF-E), and Deprescribing Benzodiazepines in Older Adults (DBO) measures.

Twenty-five articles in this scoping review were retrospective cohort studies [23,27,31,33,34,42,43,45,48, 35,44, 25, 17,24,26,29,36–41,46,47,49]. One was a mixed-method study, including a retrospective cohort study and qualitative informant group interviews gauging the opinions of clinicians and clinical care leaders [30]. Finally, there were two randomized controlled trials [28,32].

Harris et al. [17] did not provide rates for the HEDIS^®^ SUD measures [17]. All studies, except Harris et al. conducted a further analysis using the HEDIS^®^ SUD measures. Eighteen articles reported on additional non-HEDIS^®^ SUD-related metrics [23,27,33,42,43,45,48, 35,44, 25,28–30,32,36,38,41,46]. Of these, six reported on Alcohol and Other Drug (AOD) related comorbidities [27,30,33,41,42,45]. Additionally, ten reported on AOD history [28–30,33,36,38,43–45,48]. Four articles reported on AOD severity [23,44, 25,30]. Finally, one article analyzed the IET measure with statewide SUD abuse and dependence prevalence [35].

Seven articles analyzed the IET measure in terms of specific SUD outcomes [23,27,45, 25,28,32,46]. Four articles analyzed the IET measure against measures gauging improvement in severity, abstinence, or receipt of a buprenorphine prescription [25,28,32,46]. Finally, three articles reported on modified initiation and engagement measures by either extending the reporting period or increasing the number of qualifying visits [23,27,45].

Twenty-three out of the 28 articles in this study included age as a baseline characteristic [23,27,33,34,42–45, 48,25,26,28,29,32,36–41,46,47,49]. Other commonly reported baseline characteristics include gender/sex, race/ethnicity, mental health, index encounter practice setting, medical comorbidity, and substance used [23,27,33,34,42–45,48, 25,28,29,32,36–41,46,49]. It is also notable that 19 articles reported that a disproportionate number of male patients in their study population [23,27,33,42–45,48,25,28–30,32, 36–38,40,41, 46]. Regression analysis methods were used in 24 studies, with age, gender/sex, and race/ethnicity being the most commonly analyzed along with the HEDIS^®^ measure [23,27,31,33,34,42–45,48,25,28–30,32,36–41,46,47,49]. Finally, 17 studies investigated specialty SUD or mental health/psychiatric services and its relationship to the IET measure [23,27,33,34,42,43,45,48, 17,24,28,30,32, 38,40,41,46].

### Patient populations and clinical settings

IET study populations varied and included adults, adolescents, various ethnicities, and patients living with HIV [36,47,48]. Three articles investigated IET results between rural and urban populations [39,45,48]. Regression analysis results comparing the IET measure to age, gender/sex, and race/ethnicity were largely inconclusive but pointed to possible worse results in females compared to males, African Americans compared to Whites, and worse rates with age [23,27,31,33,34,42–45,48, 25,28–30,32,36–41,46,47,49].

There was variability in the clinical settings where the HEDIS^®^ SUD metrics were computed. There were studies focused on ED care [23,46], ED and primary care [37], primary care [28,32], mental health care [29,49] and specialty addiction care [27]. Our review found that individuals were more likely to engage in SUD treatment in specialty SUD and psychiatric service settings [27,33,34,42–44, 24,28–30,32,36–38,40,41].

Harris et al. conducted three studies comparing HEDIS^®^ SUD metrics between inpatient and outpatient care, and differentiated between health care specialties (SUD, psychiatric, other) [25, 17,24].

Others computed the HEDIS^®^ SUD metrics using data from beneficiaries of Medicare [30], Medicare Advantage [31,47], Medicaid [48, p. 2, 42, 35,39], and other health plans [26]. Three studies computed HEDIS^®^ SUD metrics in the Military Health System (MHS) [43,44] and the Veterans Administration (VA) [45]. The largest studies on HEDIS^®^ SUD metrics involved data from seven US health care systems [33,34,36,38–41].

### Overlaps in scope of research

Also of note, 13 articles had similar data sources, study populations, and authors. Seven of these articles used the same data from seven diverse US health systems with data from Electronic Health Records and Insurance Claims through a Virtual Data Warehouse (VDW) with similar time periods and written by overlapping authors [33,34,36–38,40,41]. Of these, four articles stated that data from 41 was used for analysis [34,36–38]. In addition, Setodji et al. [32] used data from Watkins et al. [28] and Edmonds et al. [45] used data from Bensley et al. [27,28,32,45]. Similarly, Gray et al. [44] and Adams et al. [43,44] both investigated enlisted active duty and National Guard/Reserve soldiers returning from deployment to Afghanistan or Iraq using data from an MHS data repository while having overlapping authors and study periods [43,44].

### Publications on initiation and engagement of substance Use Disorder treatment (IET)

Twenty-seven out of 28 included articles discussed IET. Of these, twenty-three articles reported an initiation rate, twenty-five articles reported an engagement rate, and twenty-two articles reported both an initiation and engagement rate [23,27,31,33,34,42–45,48, 25, 17,24,26,28–30,32,36–41,46,47].

Dates of publication for the articles ranged from the beginning of the measure in 2004 to 2022. In addition, the definition of engagement for two articles utilized the new definition of within 34 days of initiation while the remainder used an older definition of 30 days [11,42,46].

Notably, the HEDIS^®^ definition of initiation considers patients diagnosed in the inpatient setting to have initiated treatment [11,34]. Initiation results for the inpatient setting were nearly 100% or not reported in six publications [33,34, 24,36,38,40,41] and excluded from our analysis.

### Substance reporting

Seven articles examined IET in relation to specific substances, including alcohol, cannabis, and opioids [27,32,34,38,44–46]. Watkins et al. [28] and Setodji et al. [32] investigated collaborative care, IET, and self-reported abstinence, while Harris et al. [17] investigated IET and its association with clinical progress notes [17,28,32]. Harris et al. [25] investigated the relationship between engagement and the Addiction Severity Index Alcohol, Drug, and Legal composite scores [25]. Harris et al. [25], Bensley et al. [27], Urbanoski et al. [29], Englander et al. [42], and Regan et al. [46] investigated IET in relation to SUD-related treatment services [27,42, 25,29,46]. Watkins et al. [28] and Setodji et al. [32] were also randomized controlled trials [28,32]. Eight papers presented IET results by substance used [23,29,30,36–38,42,48].

### Patient-centric quality measurements

Eleven studies included patient-focused Opioid Use Disorder (OUD) metrics [22,33,34,43, 24,26,32,38,40,47,50]. Yarborough et al. discussed patient-centered care as one of five key system changes that could improve IET performance [47]. Aspects related to patient-centered care, such as patient-centered care assessments and collaborative care, were a part of the treatment models mentioned in three studies [26,37,43]. Another area of patient-centered care that was reported on was the dispensing of medications for opioid use disorder, including buprenorphine, methadone, and naltrexone, among others [43, 24,38,41]. The benefits of medications for opioid use disorder were also discussed in three articles [26,37,47].

## Discussion

Based on the findings of this literature review on HEDIS^®^ metrics for SUD, we recommend that future research on HEDIS^®^ metrics on SUD should focus on:

Include information on study populations (age, veteran status, gender/sex, race/ethnicity, mental health, and medical comorbidity), substance used, clinical setting, and geography as meaningful variables: Future research should be mindful of these variables due to their impact on the HEDIS^®^ IET measure related to SUD. These variables should be considered during study planning and data analysis. We recommend their inclusion as baseline characteristics in future studies. They are significant to help understand whether treatment is received equitably across the variable groups, help policymakers frame appropriate decisions, and decrease the disparity in treatment outcomes [23,27,33,34,42–45,48, 25,26,28,29,32,36–41,46,47,49]. These factors should also be included in any regression and sub-group analysis.

Consider intended study population characteristics and research period when selecting HEDIS^®^ metrics: Future researchers must be wary of measure specifications when selecting HEDIS^®^ metrics for their studies. NCQA methodologies are carefully developed or refined each year to ensure standardized measures remain relevant and feasible for implementation and benchmarking across all evaluated entities [19]. Thus, changes in annual methodologies must be clearly understood and discussed in longitudinal analyses. This also poses challenges for studying specific populations that may be systematically excluded per conventional methodologies. For example, instances only qualify for the IET measure denominator when the individual was enrolled with the health plan for a considerable time before and after the qualifying event. This systematically disqualifies events for justice-involved individuals who typically lose health insurance coverage during incarceration. Researchers may modify the HEDIS^®^ methodology to evaluate IET for such a population (e.g. Arizona Medicaid Targeted Investments Program Justice AOD measure [51]), but this sacrifices the ability to compare performance to other entities- even in the same year.

Include more data sources and consider medical record-sharing regulations: 13 out of the 28 identified articles had similar data sources, study populations, and authors [27,28,32–34,36–38,40,41,43–45]. Seven of these articles used the same data with similar time periods and overlapping authors [33,34,36–38,40,41]. A broader research scope is advised to study the application of SUD-related HEDIS^®^ metrics better and remove potential bias, and identify gaps in data availability. Also, discussions on the implications of SUD data sharing laws and regulations on the computation of HEDIS^®^ SUD metrics, and access to alternative sources of related data (such as registry data and ADT alerts) were missing from the publications retrieved through this literature review. For instance, Title 42 of the Code of Federal Regulations Part 2, ‘Confidentiality of Alcohol and Drug Abuse Patient Records’, allows patients to restrict provider’s access and redisclosure of SUD medical records [12–14]. On the other hand, Prescription Drug Monitoring Program (PDMP) data provide extensive information on state opioid prescriptions that could be used to support opioid-related HEDIS^®^ metrics [52].

Research HEDIS^®^ metrics different from the IET measure: While 27 out of 28 publications focused on the IET measure, studies on other SUD-related measures (HEDIS^®^ DSU, FUI, FUA, HDO, UOP, COU, POD, ASF-E, and DBO) were sparse [33,53,54, 55, 25,38,39,41,56]. Reasons for this may be the abundance of literature on IET measures and evidence associating the IET measure with clinical outcomes gauging SUD improvement [25,28,32,46]. Another reason could be challenges in tracking and reporting the information required to assess other SUD-related metrics and support learning/improvement systems. Future research on HEDIS^®^ measures beyond the IET measure is needed.

***Report on other substances besides alcohol, cannabis, and opioids:*** This scoping review identified four articles investigating alcohol, cannabis, and opioids [27,38,45,46]. While these substances cover a significant portion of substances associated with SUD, other substances that may warrant closer investigation include, but are not limited to, stimulants, benzodiazepines and hypnotics, hallucinogens, as well as nicotine [57]. More targeted research into these substances may reveal patterns that can be used to improve SUD treatment and the SUD HEDIS^®^ measures.

Investigate the role of specialty SUD and psychiatric services in SUD treatment: Individuals were more likely to engage in SUD treatment in specialty SUD and psychiatric service settings [27,33,34,42–44, 24,28–30,32,36–38,40,41]. This adds to the literature supporting these services in SUD treatment [50,58,59]. Specialty training in SUD treatment and the push for evidence-based treatment may be driving factors for these improved results.

Consider patient perspectives when determining HEDIS^®^ metrics: Eleven of the 28 studies included patient-focused Opioid Use Disorder (OUD) metrics [22,33,34,43, 24,26,32,38,40,47,50]. As Taylor Keller et al. study recommended [60]. When determining HEDIS^®^ SUD metrics, patients’ perspectives should be considered, including (1) patient experience and engagement, (2) quality of life, (3) identification of patient risks, (4) interventions to mitigate patient risks, (5) treatment; and (6) care coordination and navigation.

### Study limitations

First, since this is a scoping review, a critical appraisal and assessment of publication bias of the resulting literature were not conducted. Given the volume of literature on the IET measure, future work may include a meta-analysis to investigate factors contributing to SUD initiation and engagement. In addition, an assessment of bias may be useful to investigate the effects of overlapping data sources.

Second, our results were limited to peer-reviewed published papers. Relevant articles may have been missed, mainly if they reported on similar measures such as those from the National Quality Forum [61].

Third, relevant publications that built the foundations for introducing HEDIS^®^ measures may be missing from the literature review, when not labeled with the keywords HEDIS^®^ OR ‘Healthcare Effectiveness Data and Information Set’. Our research did not apply snowballing methods that could have led to the inclusion of those relevant published work [62–69].

## Conclusion

The outcomes of this systematic review of published, peer-reviewed publications that quantified HEDIS^®^ SUD metrics identified knowledge gaps and suggested that more research is needed on these metrics and their usefulness in informing SUD prevention and treatment, policy, and public health outcomes.

Future SUD research and public policies and intervention programs that leverage their findings must mind these gaps when selecting HEDIS^®^ measures, interpreting results, and adopting recommendations. Specifically, researchers, policy makers, and providers must consider: 1) the characteristics of the study population as applicable to sub-group trends, significant covariates, and measure parameters each year of the analysis; 2) the specificity of the measure in relation to the type(s) of substance use evaluated, nature of assessed outcome(s), and type(s) and reliability of permissible data source(s); and 3) the ubiquity of the measure across the healthcare system pertaining to the role and setting of providers (e.g. emergent, inpatient, specialists, prescribing, care managers), treatment modalities within their scope of practice, and expectations for care coordination (e.g. referral and medical sharing regulations).

## Data Availability

Data sharing is not applicable to this article as no new data were created or analyzed in this study.

## Appendix A

**Table A1.** Descriptions of healthcare effectiveness data and information set (HEDIS)^®^ measures of interest with details on NQF endorsement and CMS use.

SUD Metric	Description	NQF Endorsed	CMS Used
Diagnosed Substance Use Disorder (DSU)	Percentage of individuals ≥13 years of age diagnosed with substance use disorder. Rates reported include the percentage of members diagnosed with alcohol disorder, opioid disorder, disorder of other drugs, and substance use disorder.	NQF #2152 - Preventive Care and Screening: Unhealthy Alcohol Use: Screening & Brief Counseling (1)	No
Follow-Up After High-Intensity Care for Substance Use Disorder (FUI)	Percentage of residential treatment, detoxification visits, or acute inpatient hospitalizations that have a follow-up service for substance use disorder in individuals ≥13 years of age. Rates reported include the percentage of qualifying visits which result in follow-up within 30 days and 7 days.	NQF #3590 – Continuity of Care After Receiving Hospital or Residential Substance Use Disorder (SUD) Treatment (1)	No
Follow-Up After Emergency Department Visit for Substance Use (FUA)	Percentage of emergency department visits with a primary diagnosis of substance use disorder or drug overdose in which there is follow-up in individuals ≥13 years of age. Rates reported include the percentage of qualifying visits which result in follow-up within 30 days and 7 days.	NQF #3488 - Follow-Up After Emergency Department Visit for Alcohol and Other Drug Abuse or Dependence (1)	Adult Core Set (eff. 2017) (2) Child Core Set (eff. 2022) (2)
Initiation and Engagement of Substance Use Disorder Treatment (IET)	Percentage of new substance use disorder episodes which meet initiation and engagement criteria. Rates reported include the percentage of qualifying episodes with a qualifying encounter within 14 days (initiation) and evidence of treatment engagement within 34 days of initiation (engagement).	NQF #0004 - Initiation and Engagement of Alcohol and Other Drug Abuse or Dependence Treatment (1)	Adult Core Set (eff. 2013) (2)
Opioid Metric	Description	NQF Endorsed	CMS Used
Use of Opioids at High Dosage (HDO)	Proportion of individuals ≥18 years of age receiving prescription opioids of an average ≥90 morphine milligram equivalent for ≥15 days.	NQF #2940 – Use of Opioids at High Dosage in Persons Without Cancer	Adult Core Set (eff. 2016) (2)
Use of Opioids From Multiple Providers (UOP)	Proportion of individuals ≥18 years of age for ≥15 days from multiple providers. Rates reported include the proportion of qualifying individuals receiving opioid prescriptions from ≥4 different prescribers, ≥4 different pharmacies, as well as ≥4 different prescribers and ≥4 different pharmacies.	NQF #2951 - Use of Opioids from Multiple Providers in Persons Without Cancer	No
Risk of Continued Opioid Use (COU)	Percentage of individuals ≥8 years of age with a new episode of opioid use at risk of continued opioid use. Rates reported include the percentage of members with ≥15 days of prescription opioids in a 30-day period and ≥31 days of prescription opioids in a 62-day period.	No	No
Pharmacotherapy for Opioid Use Disorder (POD)	Percentage of individuals ≥16 years of age with an opioid use disorder diagnosis with a new opioid use disorder pharmacotherapy event lasting ≥180 days.	NQF # 3400 - Use of Pharmacotherapy for Opioid Use Disorder (OUD) (1)	Adult Core Set (eff. 2020) (2)
Alcohol Metric	Description	NQF Endorsed	CMS Used
Unhealthy Alcohol Use Screening and Follow-Up (ASF-E)	Percentage of individuals ≥18 years of age that are screened for unhealthy alcohol use and those that receive follow-up care out of the individuals with a positive screening. Rates reported include the percentage of members with a screening for unhealthy alcohol use and receiving follow-up care or counseling within 2 months of screening positive.	NQF #2152 - Preventive Care and Screening: Unhealthy Alcohol Use: Screening & Brief Counseling	No
Benzodiazepine Metric	Description	NQF Endorsed	CMS Used
Deprescribing of Benzodiazepines in Older Adults (DBO)	The percentage of individuals ≥67 years of age that are dispensed benzodiazepines and have *a* ≥ 20% decrease in diazepam milligram equivalent dose.	No	No

1.NQF: Measure Details [Internet]. [cited 2023 Oct 31]. Available from: https://www.qualityforum.org/measuredetails.aspx.

2.Medicaid. Core Set of Child and Adult Health Care Quality Measures for Medicaid (Adult Core Set), 2013–2024 [Internet]. 2024 [cited 2023 Oct 31]. Available from: https://www.medicaid.gov/sites/default/files/2023-08/2024-core-set-history-table_0.pdf.

## Appendix B

**Table B1.** Abbreviations.

AOD	Alcohol and Other Drug
aOR	Adjusted Odds Ratio
ANHOPI	Asians/Native Hawaiians/other Pacific Islanders
ASF-E	Unhealthy Alcohol Use Screening and Follow-Up
AUD	Alcohol Use Disorder
CI	Confidence Interval
COU	Risk of Continued Opioid Use
CTN	Clinical Trials Network
DBO	Deprescribing of Benzodiazepines in Older Adults
DSU	Diagnosed Substance Use Disorders
ED	Emergency Department
FUA	Follow-Up After Emergency Department Visit for Substance Use
FUI	Follow-Up After High-Intensity Care for Substance Use Disorder
HCSRN	Health Care System Research Network
HDO	Use of Opioids at High Dosage
HEDIS	Healthcare Effectiveness Data and Information Set
HIPAA	Health Insurance Portability and Accountability Act
IAD	Identification of Alcohol and Other Drug Services
IET	Initiation and Engagement of Substance Use Disorder Treatment
Medicaid MAX	Center for Medicare and Medicaid Services Medicaid Analytic Extract
MHS	Military Health System
NCQA	National Committee for Quality Assurance
NIDA	National Institute on Drug Abuse
NSUDH	National Survey of Drug Use and Health
OR	Odds Ratio
OUD	Opioid Use Disorder
PDMP	Prescription Drug Monitoring Program
POD	Pharmacotherapy for Opioid Use Disorder
PRISMA-ScR	Preferred Reporting Items for Systematic Review and Meta-Analysis extension for Scoping Review
RR	Relative Risk
SUD	Substance Use Disorder
SUPIC	Substance Use and Psychological Injury Combat
UOP	Use of Opioids from Multiple Providers
VA	Veterans’ Health Administration/Department of Veterans Affairs
VDW	Virtual Data Warehouse

## Appendix C

**Table C1.** Search terms by database.

PubMed
**Search:** ((Substance-Related Disorders[MeSH Terms] OR Substance OR Alcohol OR Opioid OR Benzodiazepine OR ‘Drug Use’ OR ‘Drug Abuse’ OR SUD OR ‘Substance Use Disorder’) AND (HEDIS OR ‘Healthcare Effectiveness Data and Information Set’))
**Academic Search Premier**
**Search:** HEDIS or (Healthcare Effectiveness Data and Information Set) **AND:**(Substance-Related Disorders) OR Substance OR Alcohol OR Opioid OR Benzodiazepine OR (Drug Use) OR (Drug Abuse) OR SUD or (Substance Use Disorder)
**Elsevier/ScienceDirect (Advanced Search)**
**Find articles with these terms:** (‘Substance-Related Disorders’ OR Substance OR Alcohol OR Opioid OR Benzodiazepine OR ‘Drug Use’ OR ‘Drug Abuse’ OR SUD) **Title, abstract or author-specified keywords:** (HEDIS OR ‘Healthcare Effectiveness Data and Information Set’)
**Medline**
**Basic Search:** (mesh.Exact(‘Substance-Related Disorders’) OR Substance OR Alcohol OR Opioid OR benzodiazepines OR ‘Drug Use’ OR ‘Drug Abuse’ OR SUD OR ‘Substance Use Disorder’) AND (HEDIS OR ‘Healthcare Effectiveness Data and Information’)
